# Hamamelis in Epistaxis of Typhoid Fever

**Published:** 1885-03

**Authors:** Wm. T. Easley


### Hamamelis in Epistaxis of Typhoid Fever.

Henry B. English, æt. 22, a railroad laborer, had been complaining of the usual symptoms of typhoid fever for several days. While sitting in his chair one afternoon he felt as though "something had exploded in his nose," which was followed by a profuse epistaxis of a violent nature. He called at my office at 4 P. M. On examination, I found blood running down over the soft palate in intermittent jets, and oozing out at the anterior nares around the temporary plugs of cotton he had placed in his nostril. I introduced some nasal plugs, freshly saturated with tannic acid, which checked the bleeding immediately and appeared to be permanent, but only proved to be temporary. I then resorted to the remainder of this class of astringents—alum, gallic acid, etc., subsulphate of iron being the only ferruginous preparation resorted to. I then left my patient, telling him that I would call again in about an hour, this being 8 P. M.

On returning, I found my patient in the same precarious condition as he was when entering my office, the subsulphate of ferrum having failed to be of any therapeutical value in this case.

I then removed the old plugs and replugged the anterior and posterior nares with freshly made ones of absorbent cotton. He assured me that he would die, for his strength was failing fast, and what assistance could be given must be rendered immediately. I examined his pulse, found them weak, irregular, and his life in great jeopardy. I remembered of hearing of the hemostatic properties of hamamelis while attending lectures. I prescribed the fluid ext. in ʒss. doses every fifteen or twenty minutes, as the emergency of the case demanded. The epistaxis was controlled immediately.

His typhoid developed in a very few days. He passed through the entire attack without a single instance of the hemorrhage returning, and was convalescing, when he had perforation of the bowel, and died before medical aid could be summoned.

The point I wish in this case to illustrate is the value of hamamelis over other astringents and styptics in this case of epistaxis.

Wm. T. Easley, M.D.

